# Placental chorioangiomatosis: a case report and literature review

**DOI:** 10.3389/fped.2025.1612488

**Published:** 2025-07-23

**Authors:** Xixi Wu, Linbo Cheng, Shimao Zhang, Yu Lu, Tao Wang, Mi Su, Qinqin Yuan, Huisheng Ge

**Affiliations:** Chengdu Women’s and Children’s Central Hospital, School of Medicine, University of Electronic Science and Technology of China, Chengdu, China

**Keywords:** placental chorioangiomatosis, complications, prenatal diagnosis, pathomechanism, ultrasound

## Abstract

The placenta is a highly specialized temporary organ during pregnancy. As the hinge of material exchange between mother and fetus, it plays a crucial role in maintaining the fetus's intrauterine life and growth period of fetus. Placental lesions or dysfunction can cause pregnancy diseases. Placental chorioangioma is a benign tumor originating from the placental with an incidence rate of 1%, whose etiology has not yet been fully elucidated. Prenatal diagnosis can usually be done by clinical ultrasound. However, placental chorioangiomatosis, as a placental choriovascular disease, is rarely reported or studied at home and abroad. Due to the unclear etiology and pathogenesis, prenatal diagnosis of placental chorioangiomatosis before early recognition of severe maternal and fetal complications during pregnancy is sparse. Therefore, patients cannot be effectively treated, and pregnancy outcomes are often poor. Herein we provide a case at our hospital and conduct a series of literature reviews around this case to further improve the understanding of placental chorioangiomatosis, and promote early recognition and early intervention.

## Introduction

As a common placental tumor, chorioangioma is a benign hamartomatous vascular tumor composed of proliferating capillaries originating from chorionic mesenchyme. Most chorioangiomas are small in size, with only about 1% detectable during histological placental examination (1). Currently, the prenatal diagnosis of chorioangioma is primarily reliant on prenatal ultrasound, which detects increased vascular flow within a placental mass distinct from the increased peripheral placental blood flow. Typically, chorioangioma is solitary, encapsulated vascular nodule that cause no maternal or fetal symptoms. Only large lesions exceeding 4cm in diameter may induce potentially fatal fetal complications such as anemia, thrombocytopenia, hydrops, and even heart failure (2–4).

Chorioangiomatosis, in contrast, refers to the diffuse, multifocal proliferation of placental capillaries within the chorionic villi, involving multiple cotyledons without well-defined margins. It is distinguished from solitary chorioangioma by its non-encapsulated, widespread microvascular pattern, absence of mass effect, and association with chronic placental insufficiency. Due to the small and scattered nature of its lesions, prenatal ultrasound diagnosis of chorioangiomatosis is considerably more challenging, and it is often only identified during pathological examination. Consequently, the risk of intrauterine fetal demise is even higher when chorioangiomatosis is present. We report a case of chorioangiomatosis associated with neonatal death.

## Case report

**Figure 1:**
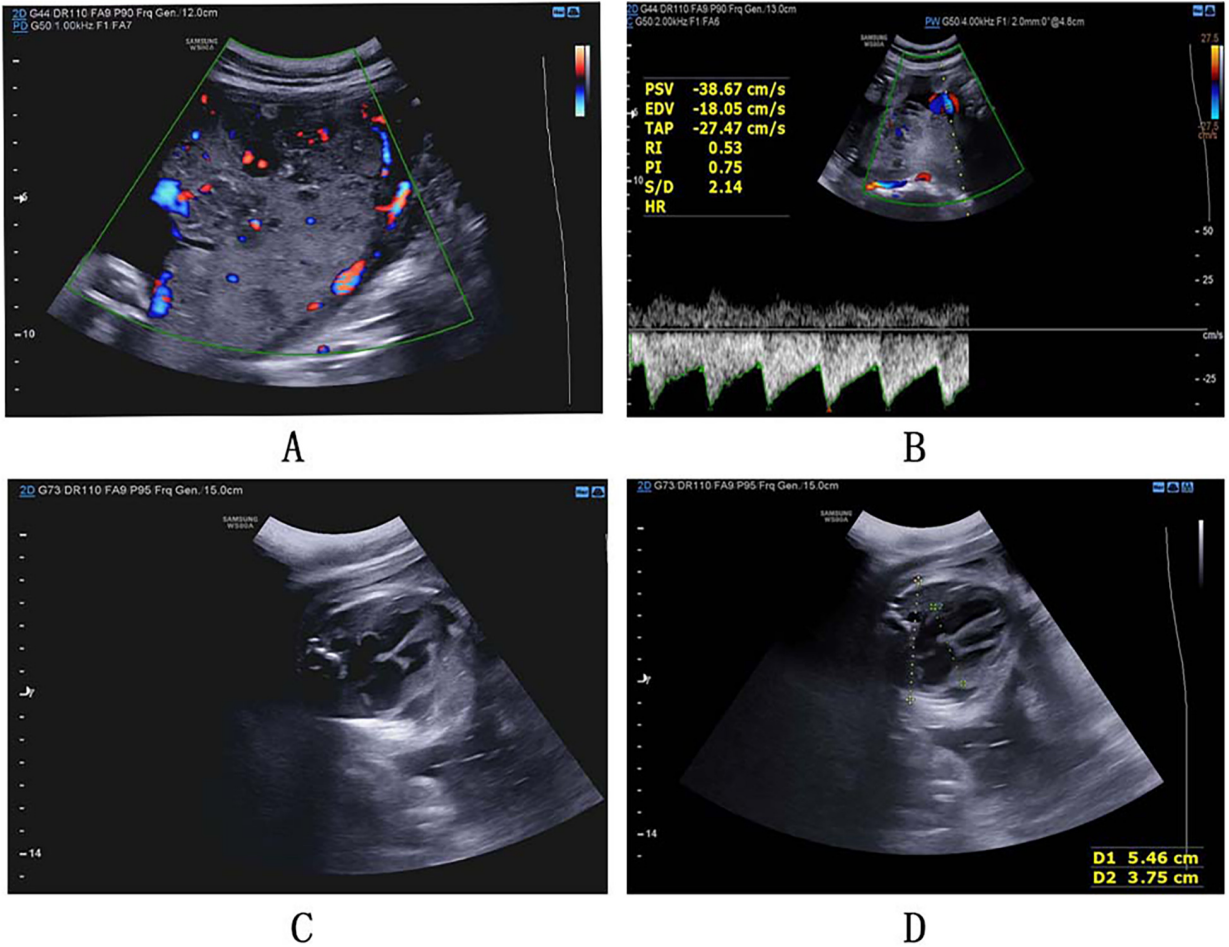
**(A)** The placenta was thickened (placenta thickness 5.7 cm), with multiple irregular low-weak echoes in the parenchyma, some of which showed honeycomb changes; **(B)** the fetal color Doppler showed that PSV was greater than 1.5 MOM; **(C)** the fetal heart grew up with pericardial effusion; **(D)** increased fetal cardiothoracic ratio.

A 26-year-old primipara who conceived spontaneously was admitted to the hospital due to potential fetal intrauterine growth restriction at 32 weeks' gestation. Her abdominal circumference and palace height were 90 cm and 29 cm, respectively. This woman has routine check-ups during pregnancy, and the check-up items for 25 weeks were normal. Fetal heart color Doppler ultrasound indicated mild tricuspid regurgitation of the fetus, and grade IV targeted fetal echocardiography showed no obvious abnormalities. The fetal four-dimensional color ultrasound prompts at the 27 + 1 weeks of pregnancy was equivalent to those at the 26 + 2 weeks of pregnancy. Patient was instructed to pay attention to balanced nutrition. The routine fetal ultrasound at 28 + 1 weeks' gestation indicated fetal development corresponding to 27 + 5 weeks, and the fetal cardiothoracic ratio was slightly larger. Until the 32 + 1 week of pregnancy, the fetus's routine ultrasound indicated that the placenta was thickened, with multiple irregular low-weak echoes in the parenchyma, some of which showed honeycomb changes. The fetal heart grew up with pericardial effusion. The size of the fetus was equivalent to that at the 30 + 2 weeks of pregnancy (Figure 1). The patient has no discomfort and the fetal movements were normal.

After admission, the fetal color Doppler examination was conducted. The result showed that the PI measurement value of umbilical artery blood flow was between 5th–50th, and the PI measurement value of fetal middle cerebral artery blood flow was also between 5th–50th. A wave of the venous catheter spectrum was not missing or vice versa, and PSV was greater than 1.5 MOM (Figure 1B). Fetal monitoring repeatedly prompted non-responsive type (variation and acceleration were not good). Considering intrauterine anemia and fetal distress, cesarean section was performed.

During the cesarean section, the uterus was seen to be 7+ months old, and a live baby girl was delivered smoothly with APGAR scores of 6-8-8 at 1, 5 and 10 min, respectively. The umbilical cord was normal, and the gas of umbilical artery showed normal pH value and base deficiency. The newborn received initial resuscitation, oxygenation of the balloon for 30 s, endotracheal intubation, and cord blood reinfusion. Then the newborn was transported to the neonatology department in an incubator for further rescue.

**Figure 2:**
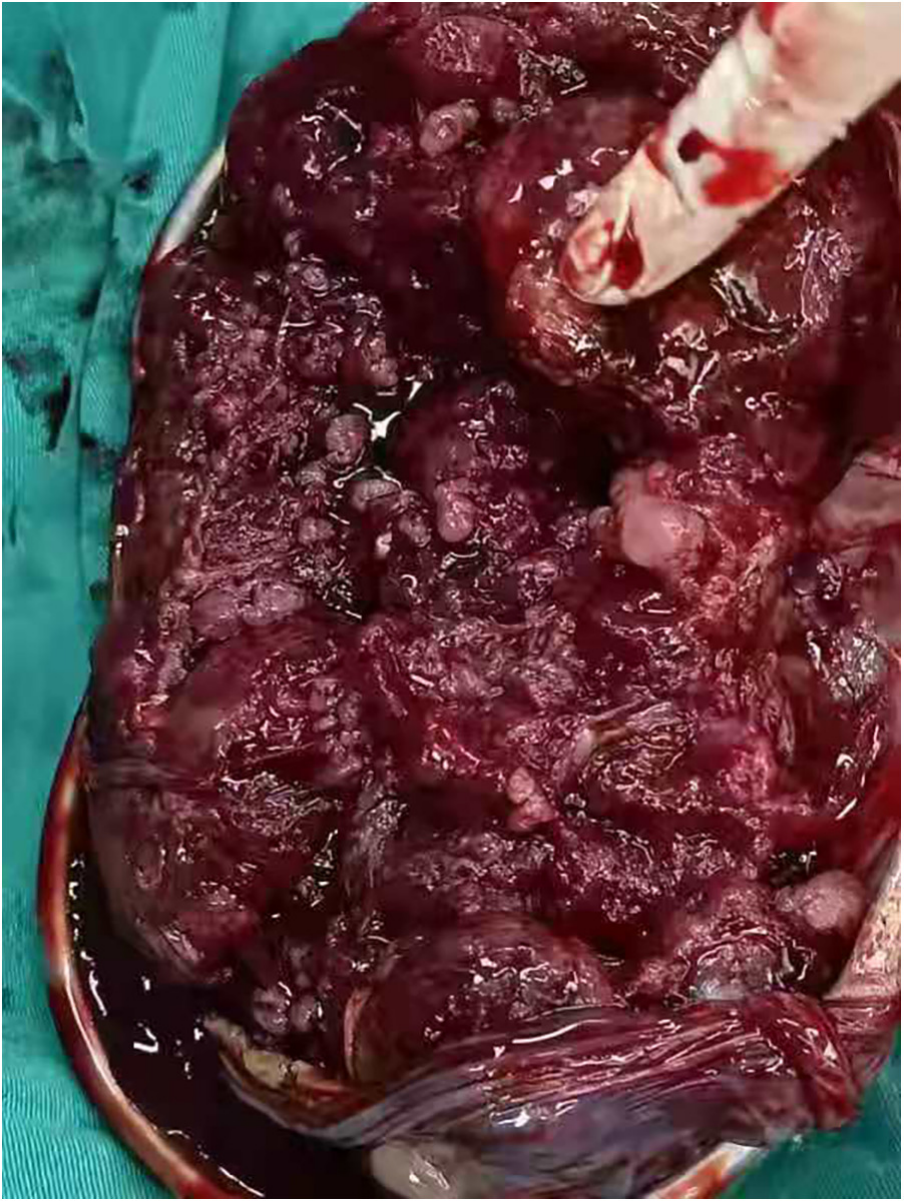
Grossly, the surface of the placenta presented a cauliflower-like protrusion (multiple small nodules of 1 mm–10 mm), and the placental parenchyma presented a dark brown soft zone with unclear boundaries.

A little amniotic fluid was sent for culture, and the placenta was sent for pathological examination. The placenta weighed 400 g and wmeasured 18 × 16 × 2 cm. The maternal surface of the placenta can be seen diffusely in the cauliflower-like changes, and the texture was brittle (Figure 2). The cesarean section proceeded smoothly, with an estimated blood loss of approximately 400 ml and a urine output of 100 ml. Maternal vital signs (including blood pressure and heart rate, etc.) remained stable and within normal limits throughout the procedure. The mother was conscious and responsive. Total recorded blood loss within the first 24 h postoperatively was 497 ml. Her recovery was uneventful and positive. She was able to ambulate on Postoperative Day 1 and had a return of bowel function, evidenced by passing flatus on the same day. In addition, the surgical wound was healing well, with no signs of infection.

**Figure 3:**
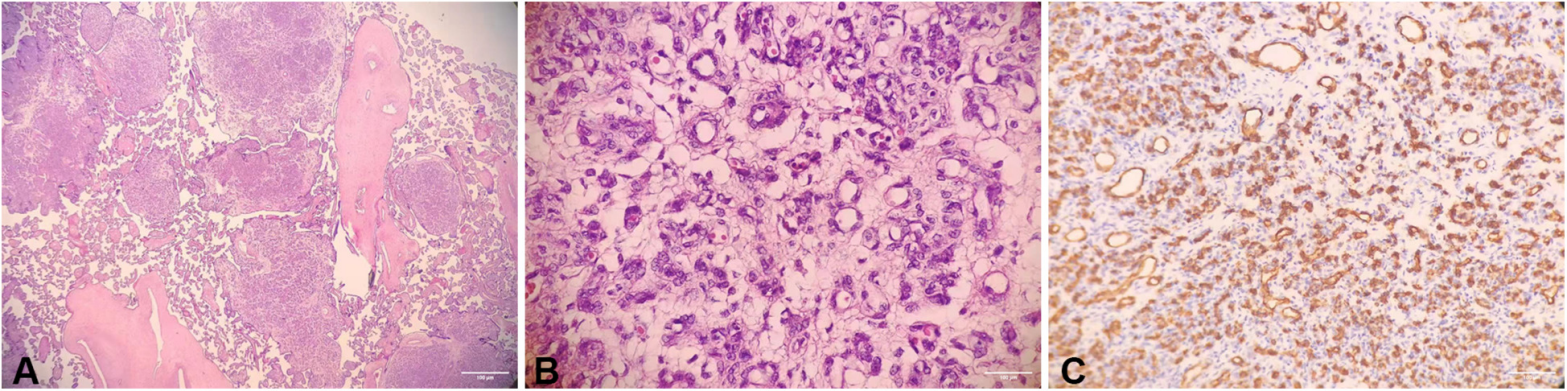
**(A)** Under low magnification (10×), the tumors were scattered among the normal placental villi with nodular; **(B)** under high magnification (40×), the small blood vessels proliferating in the tumor, the lesion only involved immature intermediate villi or stem villi; **(C)** in immunohistochemistry, CD31 (platelet endothelial cell adhesion factor-1, as a vascular-derived tumor identification factor, is highly expressed.

No obvious abnormalities were found in the results of amniotic fluid culture. The results of the pathological examination returned (Figure 3) are as follows: (placenta) single placenta, immature placenta, overgrowth of capillary vessels on multiple cuts of the placenta, and extensive involvement of placenta. Combined with immunohistochemistry, it was considered as diffuse multifocal chorioangiomatosis [vascular endothelial CD31 (+)]. There was no obvious inflammation in the fetal membranes.

Neonatal bedside cardiac color Doppler ultrasound showed: ASD, large right ventricle and large left atrium, weakened heart function (EF about 40%), mild regurgitation of mitral, tricuspid and pulmonary valves, PDA (3.6 mm, shunt right to left), pulmonary hypertension (moderate-severe); The left ventricle was D-shaped, and the inferior cavity collapse rate was less than 50%. Abdominal ultrasound showed: liver congestion.

Neonatal progress was unprosperous. After invasive ventilation treatment, meropenem actively fought against infection, blood transfusion, milrinone strengthened the heart, dopamine improved circulation, multiple blood transfusions and other supportive treatments, the newborn was still in poor conditions and died five days after delivery.

## Discussion

Placenta is a highly specialized temporary organ during pregnancy. As the hinge of material exchange between mother and fetus, it plays a crucial role in maintaining the fetus's intrauterine life and growth period. Placental lesions or dysfunction can cause pregnancy diseases. Placental chorioangioma, whose etiology has not yet been fully elucidated, is a benign tumor originating from placental with an incidence rate of 1% (1). It is generally considered to be the result of abnormal blood vessel proliferation of fibrous matrix originating from villi tissue at different stages of differentiation. 80% of the patients are asymptomatic, because their placenta has not yet reached the degree of compressing the umbilical vein or affecting the growth and development of the fetus. In other words, chorioangioma that leads to symptoms or clinically diagnosed are rare. The prenatal diagnosis of placental chorioangioma mainly relies on color Doppler ultrasound prompts, while the definite diagnosis mainly relies on postpartum placental pathological examination. However, chorioangiomatosis, defined as a diffuse proliferation of the placental capillaries permeating villous tissue, is an even rarer manifestation and needs to be differentiated from placental chorioangioma.

The vascularization of placental villi mainly undergoes two stages: the vasculogenesis stage that begins in the early pregnancy and the angiogenesis stage in the late pregnancy. Current studies suggest that chorioangiomatosis occurs in the angiogenesis stage of early pregnancy. Thus, although it has the characteristics of increased villus capillaries, it only involves immature intermediate villi or primary stem villi, and the terminal villi are generally undamaged. The blood vessels are surrounded by loose reticular fiber and merge with the surrounding interstitium. Histologically, placental chorioangiomatosis is divided into two categories: localized (focal or partial) and diffuse. Localized chorionic hemangioma lesions involve more than 5 villi, and focal chorionic hemangioma lesions involve less than 5 villi. Diffuse chorioangioma disease involves multiple independent areas that are not easily visible to the naked eye, and occasionally involves multiple small nodules that can be recognized by the naked eye (5, 6). In most cases, healthy vascular can be confirmed by color Doppler. But for chorioangiomatosis, no Doppler flow is visualized in the diseased area. This can be explained by the predominance of small capillaries, in which the flow velocities are too low to be detected, or by the occurrence of thrombosis, necrosis, fibrosis or calcifications (7–9). Hence, prenatal ultrasound is difficult to identify or capture it. However, prenatal ultrasound remains the primary diagnostic modality for placental chorioangiomatosis, particularly in patients with a prior diagnosis. Given the unpredictable clinical course, they suggested that if patients with placental chorioangiomatosis exhibit placental thickening, weekly ultrasound monitoring is advisable to diagnose fetal complications associated with an early inpatient hospitalization, and daily surveillance should be carried out at the age of previous accidents (9).

The diagnosis of chorioangiomatosis mainly depends on the pathological examination of the placenta after delivery. Placental chorioangiomatosis is characterized by the proliferation of capillaries in the villous interstitium and the disordered arrangement, and the latter instance will affect the blood supply of the placental villi and hinder the transport of glucose and oxygen from the mother to the fetus, thereby negatively affecting the mother and the fetus, such as polyhydramnios (30%), massive umbilical vein thrombosis (10), fetal hydrops (11–13), fetal distress, anemia, thrombocytopenia (13), heart failure, IUGR (30%), congenital malformations (5, 6, 14, 15), and neonatal death. The main cause of these complications is the arteriovenous shunt and hemolysis in tumor blood vessels (16). The histological classification of chorioangiomatosis has a certain influence on the complications: Limited type with premature delivery, preeclampsia and multiple pregnancy, diffuse multifocal type with extremely preterm delivery (<32 weeks), preeclampsia, intrauterine growth restriction (smaller than gestational age), giant placenta and congenital deformity (5, 17). Tumor vascularization may be an essential prognostic element because the impact of chorioangiomatosis depends more on the abundance of its vascularization than on its size (8). The tumors of diffuse chorioangiomatosis are almost very small, and some are even easy to be ignored, but their number is so large that the function of placenta is halved. The rate of intrauterine abortion, neonatal morbidity, and mortality of patients with diffuse chorioangiomatosis are significantly higher than those of patients with localized chorioangiomatosis. Some scholars believe that patients with diffuse chorioangiomatosis and localized chorioangiomatosis are not a group of lesions and should be treated differently (5, 18, 19).

Chorioangiomatosis is an extremely rare placental disease, which often causes adverse pregnancy outcomes. Intrauterine treatments such as amniocentesis for polyhydramnios, embolization for vascular shunting and laser therapy for devascularization are still under evaluation, but the severity involved in these cases often leads to fetal death (15). The selection of fetal therapies and timing of delivery should be dictated by the fetus's viability and prognosis, prioritizing stabilization of acute complications before viability, aggressive intervention to prolong pregnancy during the peri-viability period, and expedited delivery when risks outweigh benefits at advanced gestation. Therefore, early detection and timely termination of pregnancy are essential for such patients. Maternal serum alpha-fetoprotein (AFP) elevation is thought to be associated with placental diseases, including chorioangiomas and mesenchymal dysplasia. They argued that the increase of placental volume and stem villi vessels leading to the increase of surface transfer area may be the pathogenic mechanism (15). In addition, some studies believed that AFP in the fetus of patients with maternal-fetal transfusion syndrome can reach the mother through the placenta, thus significantly increasing the concentration of alpha-fetoprotein in the mother. Moreover, the level of maternal serum b-HCG may be increased in these entities (1).

In our case, when the outpatient obstetric ultrasound indicates the possibility of fetal intrauterine growth restriction, thickening of the placenta, and a slightly larger fetal cardiothoracic ratio, the maternal serum AFP and HCG can be considered, rather than expecting the fetus to be almost dying in the uterus when gestation to 32 weeks. Additionally, increasing the frequency of routine ultrasound monitoring is warranted. This includes not only fetal factors, but also assessment of parameters such as uterine artery blood flow, maternal blood flow and placental boundaries (maternal surface/edge), as these indicators can guide early identification and assessment of such lesions. Despite these instructive insights—which aim to enhance clinical recognition of placental chorioangiomatosis and reduce diagnostic errors—methodological limitations require acknowledgment: the single-case design limits generalizability; absent controls preclude comparative analysis; retrospective data risks documentation bias; and observational methods prevent causal inference. Critically, longitudinal follow-up of subsequent pregnancies in this patient could yield valuable insights into disease recurrence patterns and long-term outcomes. Nevertheless, it is hoped that this hybrid analysis lays crucial groundwork for future prospective studies on this under-characterized pathology.

## Data Availability

The raw data supporting the conclusions of this article will be made available by the authors, without undue reservation.
